# Supplementary material for: BioLORD-2023: semantic textual representations fusing large language models and clinical knowledge graph insights
Source: J Am Med Inform Assoc. 2024 Feb 27;31(9):1844–55. doi: 10.1093/jamia/ocae029 (PMC11339519; doi:10.1093/jamia/ocae029)
Supplement: ocae029_Supplementary_Data [file ocae029_supplementary_data.pdf]

APPENDIX A: NEGATIVE RESULTS

In this section, we report on alternative designs that we considered while developing our BioLORD pipeline. These results provide some insights into the limitations of our approach and suggest directions for future work.

- We initially hypothesized that generating definitions for the concepts using the knowledge graph and LLMs would replace the need for textual descriptions sampled from the knowledge graph. However, our experiments showed that the textual descriptions still contributed positively to the performance of our pipeline, as they enforced an attraction between the concepts and their parent concept names (which we used in the template). Therefore, we decided to use both the definitions and the textual descriptions in our final pipeline.

- We also investigated the effect of using different biomedical models as base models for our BioLORD models. We expected that these models would have an advantage over general-purpose language models fine-tuned on STS, as they were pre-trained on large-scale biomedical corpora. However, our results did not confirm this expectation, as biomedical models performed worse than the fine-tuned models. We attribute this to a lack of sentence understanding in the biomedical models, which led them to misunderstand definitions and overfit on shared tokens instead of semantic similarity. Thus, we concluded that base models with STS pre-training are critical for producing BioLORD models.

- Finally, we attempted to include hard mining in our pipeline. We implemented hard mining based on the ontological knowledge graph, by choosing siblings or siblings of ancestors (after ensuring they were not ancestors of the current concept themselves) as hard negatives. While hard mining improved NEL, it degraded semantics; it seems that hard mining was too aggressive and penalized concepts that were not very dissimilar. We think that a more sophisticated approach than hard mining would be needed in this case, with a margin which depends on the two concepts being compared.

APPENDIX B: DETAILS ON OUR EXPERIMENTAL SETUPS

In this section, we summarize the details of the experiments described in the results section. These details are mainly aimed at helping other researchers willing to replicate our experiments, but they might be insightful on their own to understand the scale of the work.

Contrastive phase

We leveraged our recently-upgraded hardware and ran our new contrastive phase experiments on an NVIDIA A40 GPU with 48Gb memory, up from the 32 Gb of the V100 GPU used in the BioLORD-2022 experiments. As a result, we increased the batch size from 96 to 128. We let our experiments run for about 9 days, corresponding to one epoch over our combined dataset.

This duration is very close to our previous experiments (7 days) and is the result of a careful balance between the above-mentioned hardware upgrade and our increased demand for flops. We reuse the best-performing hyperparameters from our past experiments (AdamW for the optimizer, WarmupLinear for the scheduler, 2e-5 for the learning rate, 5% of the data for the warmup window, 0.01 for the weight decay, 1 for the number of epochs, PyTorch 2.0 AMP for the mixed-precision training).

We release the code of our unsupervised contrastive training for easier replication of our results.

Self-distillation phase

We ran our self-distillation experiments on a single NVIDIA 3090 GPU, given the low memory requirements and relatively short training time required for this operation. Each training run takes about 5 hours (1 hour per epoch), with the default hyperparameters settings (AdamW for the optimizer, WarmupLinear for the scheduler, 2e-5 for the learning rate, 5% of the data for

the warmup window, 0.01 for the weight decay, 5 for the number of epochs, PyTorch 1.7.1 AMP for the mixed-precision training). We also release the code of our supervised training to facilitate the replication of our results.

Weight averaging phase

The weight averaging phase implements the Greedy Model Soup strategy described in the original paper [44]. In our experiment, we found that merging 7 closely-related fine-tuned models performed best among the configurations which were attempted. We release the resulting average weights as our final BioLORD-2023 model.

Cross-Lingual distillation

We ran our cross-lingual distillation experiments on a single NVIDIA 3090 GPU, given the low memory requirements and relatively short training time required for this operation. Each training run takes about 40 hours (4h per epoch), with the default hyperparameters settings (AdamW for the optimizer, WarmupLinear for the scheduler, 2e-5 for the learning rate, 5% of the data for the warmup window, 0.01 for the weight decay, 10 for the number of epochs, PyTorch 1.7.1 AMP for the mixed-precision training).

The training data consists of all UMLS concept names, as well as parallel translation pairs for SNOMED-CT terms in the regional languages it supports (i.e. 1428k pairs in Spanish, 703k in French, 452k in German, 1444k in Dutch, 284k in Danish, and 412k in Swedish).

We leave for a future work the usage of UMLS synonyms in languages other than English, and the addition of definitions in the distillation procedure.

APPENDIX C: DESCRIPTION OF EVALUATION DATASETS

Clinical Semantic Textual Similarity

MedSTS [46] is a dataset which was developed for evaluating clinical semantic textual similarity. It contains 1,068 sentence pairs which were annotated by two medical experts with semantic similarity scores of 0-5 (low to high similarity).

MedNLI [47] is a dataset initially developed for evaluating natural language inference reasoning over the clinical domain. It is curated by doctors tasked with providing three statements (one entailed, one contradicted, and one neutral) grounded in the medical history of a given patient. We report the proportion of hypothesis statements which are more similar to their entailed statement than their contradictory statement.

BIOSSES [48**Error! Reference source not found.**] is a biomedical semantic similarity dataset containing 100 sentence pairs and which focuses on scientific articles in the biomedical domain, rather than clinical notes. It is a challenging dataset because of the length of its entries, which often contain several sub-sentences.

SICK [49] is a dataset which consists of about 10k English sentence pairs, designed to be rich in lexical, syntactic, and semantic phenomena. Pairs have been annotated for relatedness on a 0-5 scale.

STS Benchmark [50] is a dataset which regroups several other general-purpose text similarity datasets (and contains 8628 sentence pairs). It was developed as a public benchmark for the first shared task of SemEval-2017, a workshop focusing on the evaluation of semantic models.

Biomedical Concept Representation

EHR-RelB [53] is a dataset containing 3630 concept pairs sampled from electronic health records, rated for relatedness by 3 doctors.

UMNSRS [54] is a pair of datasets, consisting of 725 clinical term pairs whose semantic similarity and relatedness were determined on a continuous scale by 4 clinicians.

MayoSRS [55] is a dataset formed by 101 clinical term pairs whose relatedness was reported on a 4-point scale by nine medical coders and three physicians.

Biomedical Named Entity Linking

TwiMed [57] provides a comparable corpus of texts from PubMed (abstracts) and Twitter (posts), allowing pharmacovigilance researchers to better understand the similarities and differences between the language used to describe disease and drug-related symptoms on PubMed (TwiMed-PM, clinical domain) and Twitter (TwiMed-TW, social media domain). Both sets of data contain 1000 samples.

SMM4H [58] is a dataset for Adverse Drug Event (ADE) normalization. It was used in the SMM4H 2020 shared task on ADE normalization. The aim of the subtask was to recognize ADE mentions from tweets and normalize them to their preferred term in the MedDRA ontology. The dataset includes 1212 tweets.

PsyTAR [59] contains patients' expression of effectiveness and adverse drug events associated with psychiatric medications, originating from a sample of 891 drugs reviews posted by patients on an online healthcare forum.

CADEC [60] is a corpus of user-generated reviews of drugs that has been annotated with adverse drug events (ADEs) and their normalization. It contains 1250 posts from a medical forum, which were annotated by a team of experts from the University of Arizona.

APPENDIX D: RESULTS FOR ALL MODELS

In the table below, the scores of two additional models (GatorTron-Base and MedCPT-Query) have been added for completion. Despite being a much larger model, GatorTron does not outperform BioLORD models for most of the tasks.

		BioSyn [9]	SapBERT [10]	GatorTron-Base [61]	MedCPT-Query [62]	BioLORD-2022	BioLORD-2023
**STS**	**MedSTS** [46]	84.0	86.0	87.9	83.5	86.3	**88.3**
	**MedNLI-S** [47]	89.5	90.5	**94.1**	87.9	89.9	92.4
	**BIOSSES** [48]	**92.1**	89.3	89.8	90.1	84.0	86.1
	**SICK** [49]	86.7	80.3	88.7	85.0	89.3	**90.3**
	**STS** [50]	79.4	81.9	84.6	78.5	86.5	**87.8**
	(average)	86.3	85.6	**89.0**	85.0	87.2	**89.0**
**BCR**	**EHR-Rel-B** [53]	42.5	51.7	50.3	59.1	57.5	**63.6**
	**UMNSRS-S** [54]	43.6	53.0	28.3	**65.0**	56.0	59.2
	**UMNSRS-R** [54]	39.1	47.5	27.3	**61.5**	54.4	54.4
	**MayoSRS-S** [55]	45.1	62.5	41.8	69.6	**74.7**	74.4
	(average)	42.6	53.7	36.9	**63.8**	60.7	62.9
**NEL**	**TwiMed-TW** [57]	42.8	48.3	30.8	**49.8**	48.5	**49.8**
	**SMM4H** [58]	33.1	43.4	22.5	41.6	46.5	**47.7**
	**PsyTAR** [59]	52.4	64.8	44.4	59.8	64.7	**66.3**
	**CADEC** [60]	35.3	40.4	27.6	42.0	58.7	**63.0**
	**TwiMed-PM** [57]	65.3	70.1	53.2	**71.4**	70.4	69.4
	(average)	45.8	53.4	35.7	52.9	57.8	**59.2**

Table D1: *Performance characteristics of state-of-the-art biomedical models on* ***STS*** *(Pearson correlation),* ***BCR*** *(Spearman correlation), and* ***NEL*** *(Top1 Accuracy). The following models are evaluated:* ***BioSyn*** *(state-of-the-art in 2020),* ***SapBERT*** *(state-of-the-art in 2021),* ***GatorTron-Base*** *(a much larger model, released in 2022),* ***MedCPT-Query*** *(a model trained using a very different dataset, released in 2023),* ***BioLORD-2022*** *(our baseline), and* ***BioLORD-2023*** *(our new model). Bolding and a color code indicate the best and second-best results for a given task.*

APPENDIX E: QUALITATIVE ANALYSIS OF RESULTS

While the quantitative results presented so far seem to confirm the strength of our new model, quantitative results hardly ever tell the full story.

By design, practically all clinical NEL datasets suffer from annotation issues, as there is often no “perfect” gold label which fully describes a particular reality, and arbitrary choices have to be made in case of ambiguity.

While this should rarely affect the ranking of different models, as the arbitrary nature of these choices cancel each other out on the long run, this makes the use of a qualitative analysis important to understand not only the number of true errors each model is doing, but also their nature. Not all errors have the same gravity and impact on patients, or on the results of retrospective studies.

In this section, we review the NEL results of 200 randomly-sampled entity mentions from the PsyTar dataset [59] covering symptoms of psychological affections and adverse reactions to their treatments, in the words of patients themselves, as reported on an online healthcare forum.

Each model answer was considered and classified in 5 categories:

- **Exact match** (when the label predicted by the model matched the gold label of PsyTar; these predictions contribute to the quantitative score).
- **Correct match** (when the label predicted by the model could have been a valid gold label for the considered input, for example because multiple MedDRA codes represent the described symptom).
- **Partially incorrect match** (when the label predicted by the model describes an affliction that's compatible with the considered input, but whose granularity is not entirely correct, for example because the severity or exact nature of symptoms is not well described).
- **Contextually incorrect match** (when the predicted label is incorrect for the considered context but can explained because of the polysemic nature of words either in the considered input or in the chosen label).
- **Incorrect** (otherwise).

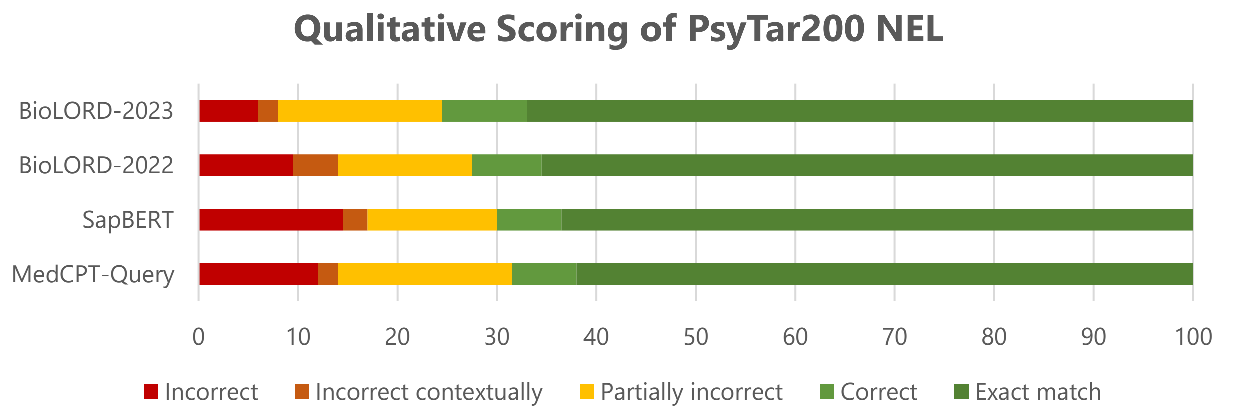

Input	SapBERT	R1	MedCPT	R2	BioLORD-2022	R3	BioLORD-2023	R4	Gold Label
anxiety had increased	Anxiety	5	Anxiety	5	Anxiety	5	Anxiety attack	3	Anxiety
affect my job	Problems at work	4	Leaving job	0	Loss of job	0	Problems at work	4	Restricted work performance
spaciness	Spasm	0	Spinal stenosis	0	Spasm	0	Tedium vitae	0	Dissociative trance
increased anxiety , only for fi	Anxiety attack	3	Anxiety	5	Severe anxiety (panic)	3	Anxiety attack	3	Anxiety
hair loss	Loss of hair	5	Loss of hair	5	Loss of hair	5	Loss of hair	5	Loss of hair
increased headaches frequer	Headache	5	Headache	5	Tension-type headache	3	Tension-type headache	3	Headache
volcano of negative emotion	Racing thoughts	3	Racing thoughts	3	Hypomanic mood	0	Emotional upset	3	Negative automatic thoughts
blurred vision	Blurring of visual image	5	Blurring of visual image	5	Blurring of visual image	5	Blurring of visual image	5	Blurring of visual image
clammy feeling	Callous character	0	Clammy sweat	5	Clammy sweat	5	Clammy sweat	5	Clammy sweat
hurt so bad	Severe pain	4	Bad taste in mouth	3	Aching sensation quality	3	Severe pain	4	Pain
severe cotton mouth	Bad taste in mouth	0	Trismus	0	Xerostomia	5	Herpes labialis	0	Xerostomia
eye twitching	Twitching eye	5	Twitching eye	5	Twitching eye	5	Twitching eye	5	Twitching eye
brain has become numb	Numbness	1	Numbness	1	Numbness	1	Numbness	1	Emotionally detached
missing meetings	Missed period	0	Missed period	0	Absenteeism at work	5	Absenteeism at work	5	Absenteeism at work

The figure above summarizes our findings, which do not differ significantly from the quantitative numbers obtained on the benchmark, but from which we can draw a few new conclusions:

1. The quantitative benchmark is a very good proxy for the model quality, with about 90% of correct answers matching the gold label for all evaluated models.
2. The quantitative benchmark does not, however, provide a very good vision into the number of truly incorrect predictions made by each model. While BioLORD improves the number of exact matches over its competitors by a few points only, it reduces the number of incorrect matches by half over MedCPT and by a two-thirds over SapBERT.

We attribute this to the increased semanticity of BioLORD-2023's latent space, which vastly reduces the occurrence of totally unrelated predictions, unlike models like SapBERT which perform very well within their training boundaries but might fail to generalize to previously unseen synonyms and paraphrases.

To visualize this better, we drew Sankey diagrams to demonstrate how the predictions of individual test cases evolve from one model to another.

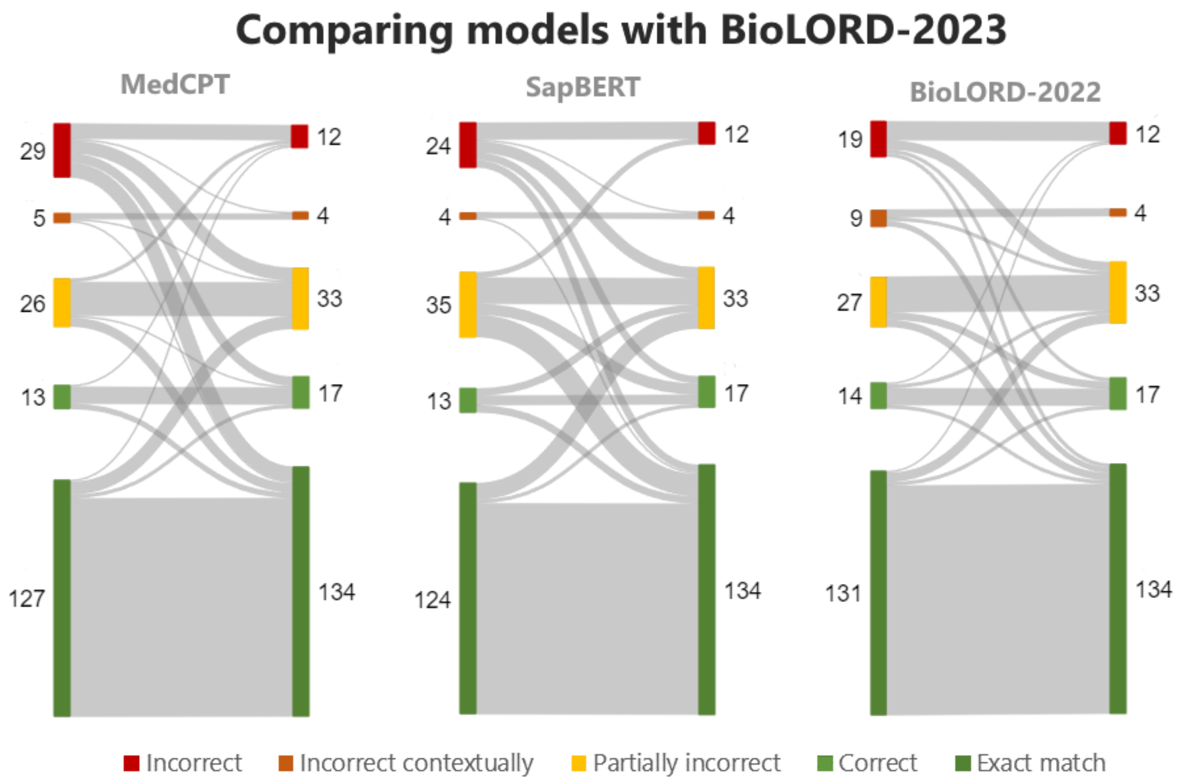

The Sankey diagrams are very insightful, because they show that most of the 16 (12+4) remaining errors of BioLORD-2023 are shared by all models, showing limited potential for ensembling (at least in the context of error reduction).

There is also close to no cross-pollination between contextually incorrect predictions and incorrect predictions, indicating that it is indeed the lack of sufficient context that throws the models off, and not a general issue which could not be fixed by better benchmarks.

The diagrams also clarify the mechanism through which BioLORD-2023 improves its ranking over other models, mainly managing to make fewer errors (high incorrect to correct cross-pollination) while not introducing new types of errors (low correct to incorrect cross-pollination).

In conclusion, BioLORD-2023 is a particularly strong clinical NEL model, especially in cases where granularity errors are not critically important, as its mistakes consist mostly of partial errors, with very few wrong classifications.
